# Identification of a Large Family of Slam-Dependent Surface Lipoproteins in Gram-Negative Bacteria

**DOI:** 10.3389/fcimb.2017.00207

**Published:** 2017-05-31

**Authors:** Yogesh Hooda, Christine C. L. Lai, Trevor F. Moraes

**Affiliations:** Department of Biochemistry, University of TorontoToronto, ON, Canada

**Keywords:** gram-negative bacteria, surface lipoproteins, outer membrane transporters, bacterial gene clusters, protein translocation pathways, flow cytometry

## Abstract

The surfaces of many Gram-negative bacteria are decorated with soluble proteins anchored to the outer membrane via an acylated N-terminus; these proteins are referred to as surface lipoproteins or SLPs. In *Neisseria meningitidis*, SLPs such as transferrin-binding protein B (TbpB) and factor-H binding protein (fHbp) are essential for host colonization and infection because of their essential roles in iron acquisition and immune evasion, respectively. Recently, we identified a family of outer membrane proteins called Slam (Surface lipoprotein assembly modulator) that are essential for surface display of neisserial SLPs. In the present study, we performed a bioinformatics analysis to identify 832 Slam related sequences in 638 Gram-negative bacterial species. The list included several known human pathogens, many of which were not previously reported to possess SLPs. Hypothesizing that genes encoding SLP substrates of Slams may be present in the same gene cluster as the Slam genes, we manually curated neighboring genes for 353 putative Slam homologs. From our analysis, we found that 185 (~52%) of the 353 putative Slam homologs are located adjacent to genes that encode a protein with an N-terminal lipobox motif. This list included genes encoding previously reported SLPs in *Haemophilus influenzae* and *Moraxella catarrhalis*, for which we were able to show that the neighboring Slams are necessary and sufficient to display these lipoproteins on the surface of *Escherichia coli*. To further verify the authenticity of the list of predicted SLPs, we tested the surface display of one such Slam-adjacent protein from *Pasteurella multocida*, a zoonotic pathogen. A robust Slam-dependent display of the *P. multocida* protein was observed in the *E. coli* translocation assay indicating that the protein is a Slam-dependent SLP. Based on multiple sequence alignments and domain annotations, we found that an eight-stranded beta-barrel domain is common to all the predicted Slam-dependent SLPs. These findings suggest that SLPs with a TbpB-like fold are found widely in *Proteobacteria* where they exist with their interaction partner Slam. In the future, SLPs found in pathogenic bacteria can be investigated for their role in virulence and may also serve as candidates for vaccine development.

## Introduction

Gram-negative bacteria contain an asymmetric outer membrane that protects the bacteria from environmental stress and acts as a shield from harmful chemicals (Silhavy et al., 2010). However, this creates a logistic challenge for the transport of various biomolecules to and from the extracellular milieu and across the outer membrane of the bacteria (Pagès et al., 2008; Delcour, 2009). Toward this end, Gram-negative bacteria have developed dedicated translocation systems that deliver various families of proteins and other biomolecules across the outer membrane (Nikaido, 2003; Costa et al., 2015; Geyter et al., 2016). The mechanism for this transport is distinct from translocation systems found in the inner membrane owing to the lack of ATP in the periplasm and is an active field of research (Karuppiah et al., 2011).

Surface lipoproteins or SLPs are a class of soluble proteins that are present on the surface of Gram-negative bacteria. They are anchored to the outer membrane via three fatty acyl chains that are post-translationally attached to their N-termini (Wilson and Bernstein, 2016). The first reported SLP was TraT, a protein of the F sex factor in *E. coli* (Manning et al., 1980). Several other SLPs were identified soon after within a few Gram-negative bacterial families including *Klebsiella* (Pugsley et al., 1986), *Neisseria* (Schryvers and Morris, 1988), and *Spirochetes* (Chamberlain et al., 1989; Brandt et al., 1990). Recently, there has been an increase in the number of reports of SLPs from different bacterial species with distinct structural folds and surface topologies (Konovalova and Silhavy, 2015). SLPs are involved in several important cellular pathways for nutrient acquisition, cellular adhesion and stress response (Zückert, 2014; Szewczyk and Collet, 2016; Wilson and Bernstein, 2016).

The discovery of SLPs in different bacteria has raised questions regarding the biosynthetic pathway used by these proteins for their synthesis and transport to the surface. SLPs are synthesized in the cytoplasm and transported to the periplasm by the Sec or Tat machinery based on the signal sequence present on the SLPs (Chatzi et al., 2013). Once in the periplasm, three enzymes in the inner membrane process the SLPs by cleaving the signal sequence and attaching three fatty acyl chains to the N-terminal cysteine residue (Szewczyk and Collet, 2016). Upon lipidation, most SLPs are transported across the periplasm to the inner leaflet of the outer membrane through the Lol system (Okuda and Tokuda, 2011). However, there are a few exceptions to this rule, including pullulanase that avoids the Lol system and moves to the surface through the Type-II secretion system (D'Enfert et al., 1987). Additionally, in *Borrelia* sp., SLPs are proposed to require a periplasmic “holding” chaperone that prevents premature folding of SLPs before reaching the outer membrane (Chen and Zückert, 2011; Zückert, 2014).

Upon insertion into the outer membrane, the translocation systems required for the movement of SLPs across the outer membrane remain poorly characterized. The first SLP for which the export pathway was characterized was pullulanase in *Klebsiella* sp. that utilizes the Type II secretion system (D'Enfert et al., 1987). More recent studies have shown that NalP (a neisserial SLP) functions as a Type Va “autotransporter” secretion system (Van Ulsen et al., 2003), while BamC (Webb et al., 2012) and RscF (Cho et al., 2014; Konovalova et al., 2014) in *E. coli* use the Bam complex to move across the outer membrane. Functional and mutagenesis studies in *Borrelia* sp. (Schulze et al., 2010; Chen and Zückert, 2011) and *Bacteroides* sp. (Lauber et al., 2016) have shown that the sorting rules used by these SLPs are distinct from other SLPs, indicating that different bacterial species may possess different translocation systems for the delivery of SLPs. Additionally, within *Neisseria* sp., *two* distinct SLP export pathways have been reported (Hooda et al., 2017), suggesting that multiple systems for the export of SLPs may exist in a single bacterial species.

The SLPs found in the genus *Neisseria* are amongst the most extensively studied SLPs. *N. meningitidis* and *N. gonorrhoeae* encode multiple SLPs that are involved in a variety of cellular pathways critical for survival of neisserial pathogens in humans (Hooda et al., 2017). In *N. meningitidis*, eight SLPs have been well-characterized, of which three [transferrin-binding protein B (TbpB) (Schryvers and Morris, 1988), lactoferrin-binding protein B (LbpB) (Pettersson et al., 1998) and hemoglobin-haptoglobin utilization protein (HpuA) (Lewis et al., 1997)] are involved in iron acquisition while two others: factor-H binding protein (fHbp) (Madico et al., 2006) and neisserial heparin binding antigen (NHBA) (Serruto et al., 2010) are involved in immune evasion. Other neisserial SLPs include *Neisseria* autotransporter protease (NalP) (Van Ulsen et al., 2003), anaerobically induced protein A (AniA) (Hoehn and Clark, 1992) and macrophage infectivity potentiator (MIP) (Leuzzi et al., 2005) which play roles in extracellular proteolysis, anaerobic growth and intracellular survival respectively. These SLPs have been shown to bind to different human factors and atomic resolution full-length or partial structures of these SLPs have aided in understanding their mechanism of action (Hooda et al., 2017). Recently, we described a family of outer membrane proteins called Slam or Surface lipoprotein assembly modulator that is essential for surface display of a subset of neisserial SLPs (Hooda et al., 2016). *N. meningitidis* contains two Slam proteins: Slam1 is necessary for the display of TbpB, LbpB, and fHbp, whereas Slam2 is specifically required for the SLP HpuA. Furthermore, Slam have been shown to potentiate the functional display of neisserial SLPs on the surface of laboratory strains of *E. coli* that do not possess any Slam or SLP homologs (Hooda et al., 2016). This work suggested that a subset of neisserial SLPs utilize a unique mechanism to get to the cell surface that is dependent on the Slam family of outer membrane proteins.

In our previous work, we discovered that genes encoding Slam2 and HpuA are adjacent to each other in multiple neisserial genomes (Hooda et al., 2016). Based on this observation, we searched and annotated genes upstream and downstream of 353 putative Slam homologs found in other proteobacterial species. This dataset showed that a large number of Slam related sequences are located adjacent to genes that encode putative lipoproteins with TbpB-like folds suggesting a genetic linkage between these two families of proteins. The bioinformatics analysis allowed us to identify TbpB-like SLPs in many bacterial species that were previously not known to possess SLPs, including the human pathogens *Acinetobacter baumannii* and *Salmonella enterica* subsp. *arizonae*.

## Materials and methods

### Identification of slam homologs

To generate a database of Slam related sequences, iterative psi-blast searches were performed (March 4, 2016) against a non-redundant database containing all partial and complete bacterial genome sequences using the sequence of Slam1 protein (NMB0313) from *Neisseria meningitidis* strain MC58 as the query. Four independent psi-blast searches were performed for different clades of proteobacteria (alpha-, beta-, gamma-, and delta/epsilon/zeta-proteobacteria). The lists of putative Slam genes obtained from these four psi-blast searches were pooled and only unique representative Slam sequences were kept from a given bacterial species. The list was manually checked to remove the following: (i) partial sequences (containing premature stop codons or with partial gene sequence coverage), and (ii) sequences coding for only the N-terminal domain (Ntd) of Slam. This gave a final list (Supplementary Data 1) of 832 Slam sequences spanning 638 bacterial species.

To understand the distribution of Slam related sequences, a phylogenetic tree of different proteobacterial species was made using the 16S-RNA sequences obtained from the database Greengenes (DeSantis et al., 2006). One representative member was kept from each family of bacteria. In total 52 species (8: alpha-, 10: beta-, 23: gamma-, 5: delta-, 5: epsilon-, and 1: zeta-proteobacteria) were selected for the final tree. The tree was made using the PhyML plugin in the software Geneious (Kearse et al., 2012) with 100 bootstraps. The nodes were kept if they appeared in 60% of the bootstrap runs. The presence of Slam related sequences was mapped on the phylogenetic tree.

### Analysis of gene neighborhoods around putative slam homologs

The list of 353 Slam related sequences generated in our previous study (Hooda et al., 2016) was used to further investigate the neighboring genes in the Slam gene clusters. This number is more than a third of the total Slam related sequences and covers all major bacterial phyla that possess Slam related sequences, except for epsilon- and zeta-proteobacteria (Supplementary Data 1). In selecting genomes for a given bacterial species, fully sequenced reference genomes were given preference. For each of the Slam related genes present in these species, the corresponding genomic record (NCBI genome) was used to identify genes upstream and downstream along with their corresponding functional annotations (NCBI protein database, Ensembl bacteria). In a few of the cases, no genes were predicted upstream or downstream as the Slam related genes were close to the beginning or the end of the contig respectively and these sequences were ignored.

Within the Slam related gene clusters, a number of the neighboring genes were predicted to encode lipoproteins (predicted by an N-terminal lipobox motif using LipoP and/or SignalP) and we also found many examples of genes encoding TonB dependent transporters (IPR000531). The putative lipoproteins were annotated as either GNA1870-related lipoproteins, TBP-like solute-binding proteins or pagP-beta barrel proteins (InterPro signature; IPR01490; IPR001677; IPR011250 respectively). All the genes with one of the above-mentioned annotations are included in Supplementary Data 2.

### Bacterial strains and growth conditions

Strains used in this study are summarized in Supplementary Table 1. *E. coli* were grown in LB media containing antibiotics when necessary (50 μg/mL kanamycin and 100 μg/mL ampicillin). Cloning procedures were carried out using *E. coli* MM294 competent cells. Protein expression was performed using *E. coli* C43 (DE3) cells for all the flow-cytometry and western blot analysis.

### Generation of plasmids for expression of slams and SLPs

For flow cytometry experiments, the three SLPs (*Haemophilus influenzae* TbpB, *Moraxella catarrhalis* TbpB and *Pasteurella multocida* PM1514) were cloned into pET52b (to make pET52b *Hinf* TbpB, *Mcat* TbpB or *Pmul* SLP) using the restriction-free (RF) cloning strategy (van den Ent and Löwe, 2006). The *tbpb* genes were amplified from the genomes of *H. influenzae* strain 86-028NP and *M. catarrhalis* strain O35E, and the *pm1514* gene was amplified from *P. multocida* strain h48. A FLAG tag was inserted on the C-terminus of *M. catarrhalis tbpb* using FastCloning (Li et al., 2011) to make pET52b Mcat TbpB-flag. pET52b PmSLP-flag and pET52b PmSLP-flag-Slam was cloned by replacing the *catarrhalis tbpb* gene with *pm1514* and *pm1515-pm1514* respectively in frame with the FLAG tag using RF cloning.

The corresponding Slams were inserted into pET26b (pET26 *Hinf* Slam1, *Mcat* Slam1, and *Pmul* Slam) using RF cloning (van den Ent and Löwe, 2006). A 6xHis-tag was inserted between the *pelB* and the mature Slam sequences.

### Flow cytometry

For the *E. coli* translocation assays, the display of an SLP was determined using flow cytometry. Pairs of SLP and Slam plasmids (shown in Supplementary Table 1) were transformed into C43 (DE3) cells and grown in 1 mL of auto-induction media (Studier, 2005) for 18 h at 37°C. *H. influenzae* Slam showed poor expression when grown overnight in autoinduction media. Hence, for *H. influenzae* TbpB flow cytometry assays were performed by growing cells at 37°C to an OD_600_ ~ 0.6 and then inducing protein expression by the addition of 1 mM isopropyl β-D-1-thiogalactopyranoside (IPTG). Upon induction, cells were grown at 18°C for 16–18 h. Cells were harvested, washed twice in PBS containing 1 mM MgCl_2_, and incubated with α-Flag antibodies (1:200, Sigma), or biotinylated human transferrin (0.05 mg/ml, Sigma) for 1 h at 4°C. The cells were then washed twice with PBS containing 1 mM MgCl_2_ and then labeled with R-phycoerythrin (R-PE) conjugated Streptavidin (0.5 mg/ml, Cedarlane) or R-PE conjugated α-mouse IgG (25 μg/mL, Thermo Fisher Scientific) for 1 h at 4°C. Following staining, cells were fixed in 2% formaldehyde for 20 min and further washed with PBS containing 1 mM MgCl_2_. Flow cytometry was performed with a Becton Dickinson FACSCalibur and the results were analyzed using FLOWJO software. Mean fluorescence intensity (MFI) from at least three biological replicates were used to compare surface exposure of a given SLP between different samples. Statistical significance was calculated by comparing MFI between different samples using the one-way ANOVA test available in the software Prism 6.

Western blots were used to test the expression levels of each of the constructs used for the flow cytometry experiments. α-Flag (1:5,000, Sigma) and α-His (1:5,000, Thermo Fisher Scientific) antibodies were used to test expression of the SLP and Slam constructs respectively. α-GroEL (1:10,000) antibodies were used as loading controls.

### Sucrose density ultracentrifugation

*E. coli* C43(DE3) cells expressing pET52b PmSLP-flag and pET26b empty or pET26 PmSlam were grown overnight and then used to inoculate 50 ml LB with the appropriate antibiotic. The cells were grown to an OD_600_ ~ 0.6, induced with 1 mM IPTG and then grown for an additional 18 h. The cells were pelleted, resuspended in 20 mM Tris pH 8.0, 200 mM NaCl with fresh lysozyme (1 mg/ml), 2 mM PMSF and DNase I (0.05 mg/ml), lysed by sonication and then centrifuged at 10,000 r.c.f. to remove cell debris. The supernatant was centrifuged at 125,000 r.c.f. for 1 h to collect the cellular membranes. The membrane pellet was resuspended in 1 ml of 20 mM Tris pH 8.0, 200 mM NaCl using a micro-glass homogenizer.

The inner and the outer membrane of *E. coli* were separated using a modified sucrose density ultracentrifugation protocol that was previously described (Hooda et al., 2016). For this assay, 100 μl of the membrane pellet was applied on top of a 13.2 ml thin-wall polypropylene tube containing step gradients of 3 ml of 2.02 M, 6 ml of 1.44 M and 3 ml of 0.77 M sucrose. The tubes were centrifuged at 83,000 r.c.f. for 16 h. The outer membrane and inner membranes partitioned to the interface of the 2 M and 1.44 M sucrose cushions and 1.44 M and 0.77 M sucrose layers, respectively. Twelve 1 ml fractions were collected and subjected to SDS–PAGE followed by western blotting with α-Flag (1:10,000), α-LepB (1:10,000), and α-OmpA (1:40,000) antibodies.

## Results

### Identification of putative slam homologs in gram-negative bacteria

**Figure 1:**
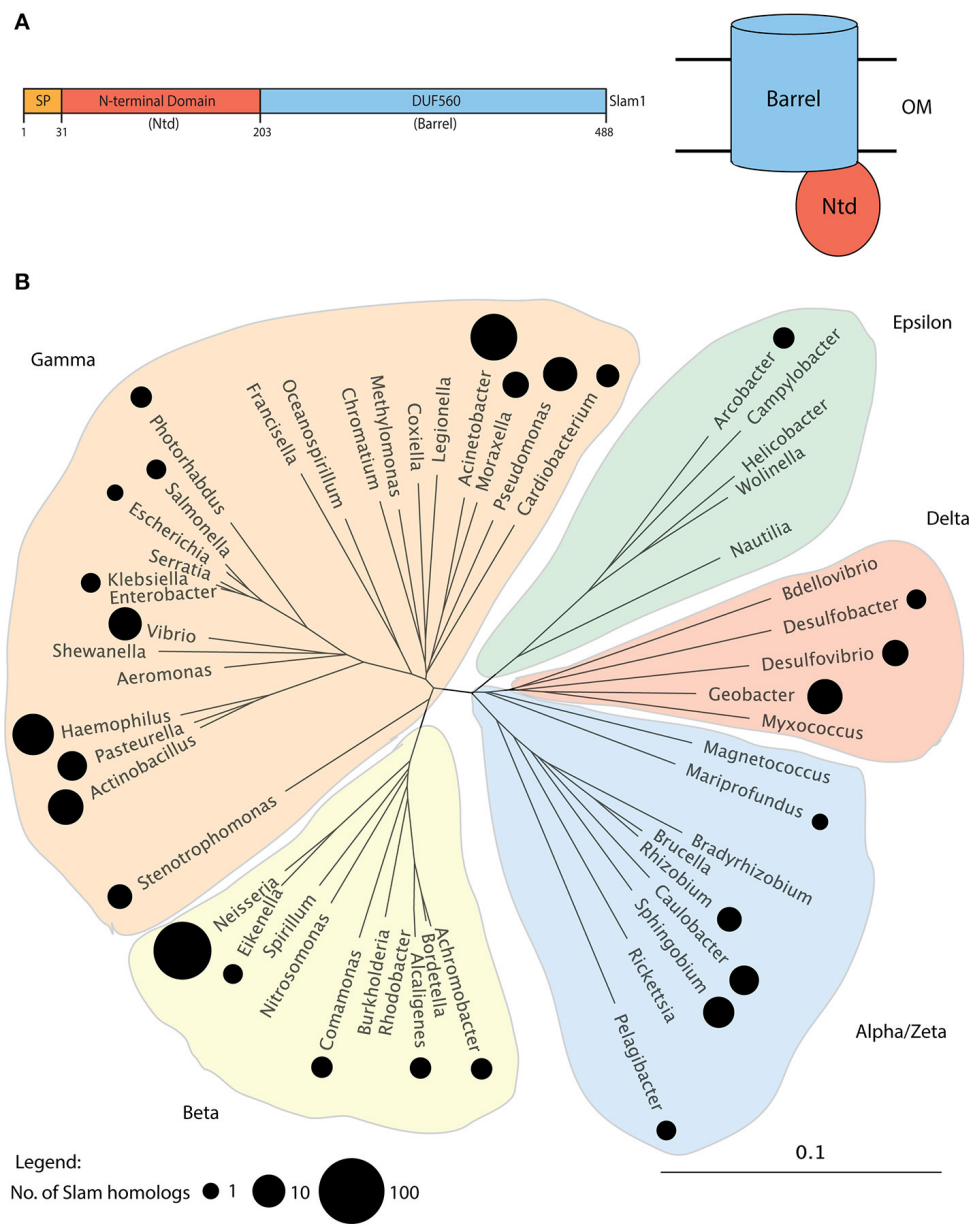
**Putative Slam family of proteins in Gram-negative bacteria**. **(A)** Domain architecture of *N. meningitidis* Slam1. Slams possess 2 domains: a periplasmic N-terminal domain (Ntd) containing tetratricopeptide repeats and a predicted membrane bound 14-stranded barrel domain referred to as DUF560. **(B)** Distribution of Slam related sequences in *Proteobacteria*. A family tree of *Proteobacteria* was made using 16S-RNA sequences from 52 species representing the major bacterial families within *Proteobacteria*. The families containing at least 1 species with a Slam related sequence containing both the Ntd and DUF560 domains are highlighted by black circles. The size of the circle represents the number of Slams identified in a given bacterial family. Slams were found within all clades of *Proteobacteria*.

In a previous study, we had used the *N. meningitidis* Slam1 sequence to perform psi-blast searches (Altschul et al., 1997) and identified 353 putative Slam homologs in 225 Gram-negative bacteria (Hooda et al., 2016). All identified Slam related sequences possessed an N-terminal domain (Ntd) predicted to contain tetratricopeptide repeats(TPR) and a C-terminal beta-barrel domain annotated as a DUF560 domain (Figure 1A). Since that study, a large number of bacterial genomes have been sequenced by next-generation sequencing techniques. Hence, we performed updated psi-blast searches and were able to identify 832 Slam related sequences in 638 Gram-negative bacteria (Supplementary Data 1). The Slam1 gene (*nmb0313*) was used as the search template and we manually analyzed the list to remove genes for which only partial sequences were available. We also removed several hits that contained a large single domain with TPR repeats that are similar to the TPR repeats found in Slam-Ntd. As was previously observed, all Slam sequences obtained in our dataset contained both the Ntd and DUF560 domains and no sequences containing only the DUF560 were obtained. With these additional genomic sequences, we were able to identify Slam related sequences in all clades of the phylum *Proteobacteria* (Figure 1B). Slam related sequences were identified in bacterial species living in diverse environments including free-living, commensal and/or pathogenic bacteria. Slam-like proteins are predicted to be found in many human pathogens such as *Vibrio cholerae, Salmonella enterica* subsp. *arizonae*, and *Acinetobacter baumannii*.

### Slams adjacent to TbpB in *M. catarrhalis* and *H. influenzae* translocate their respective TbpBs to the surface in *E. coli*

**Figure 2:**
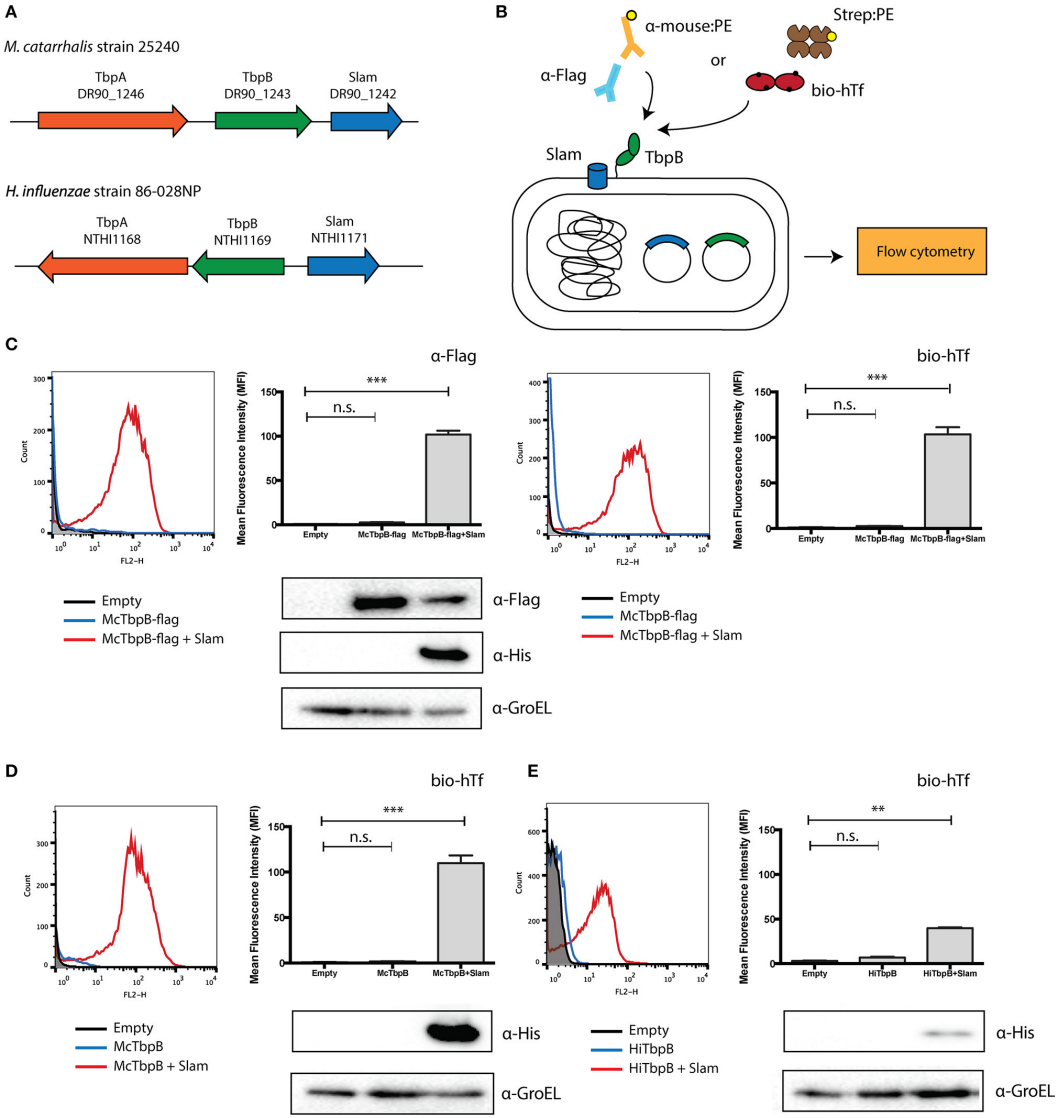
**Translocation assay with Slam and TbpB pairs from *Moraxella catarrhalis* and *Haemophilus influenzae*. (A)** Slam and TbpB gene cluster in *M. catarrhalis* and *H. influenzae*. From the bioinformatics analysis, Slam genes were found adjacent to the known transferrin binding surface lipoprotein TbpB in the human pathogens *M. catarrhalis* and *H. influenzae*. **(B)** *E. coli* translocation assay used in this study. The Slam and TbpB genes were expressed in *E. coli* C43 (DE3) cells. The cells were labeled with either biotinylated human transferrin and streptavidin linked to R-PE, or mouse α-Flag antibody and α-mouse linked to R-PE. Surface display of TbpB was quantified using flow cytometry. **(C)** Flow cytometry profiles of C43 cells transformed with empty plasmid (black), *M. catarrhalis* TbpB-flag (blue), *M. catarrhalis* TbpB-flag + Slam (red) were obtained using α-Flag (left panel) and biotinylated human transferrin (right panel). An increase in fluorescence intensity was observed in the presence of Slam for both α-Flag and biotinylated human transferrin. Surface TbpB was quantified using mean fluorescence intensity. Western blots using α-Flag and α-His were used to quantify McTbpB and McSlam. α-GroEL is used as the loading control **(D)** Flow cytometry profile of TbpB homologs from *M. catarrhalis* (McTbpB) and **(E)** flow cytometry profile of *H. influenzae* (HiTbpB) using biotinylated human transferrin. Scatter plots of cell counts (Count) vs. R-PE fluorescence (FL2-H) in the presence (shown in red) or absence (shown in blue) of Slam are illustrated in the left panels. C43 cells transformed with empty plasmid (shown in black) were used as negative controls. Surface TbpB was quantified using mean fluorescence intensity (right panels). Western blots were done with α-His to examine Slam expression and α-GroEL was used as the loading control. Statistical significance was determined using one-way ANOVA. ^***^p ≤ 0.001, ^**^p ≤ 0.01, n.s. *p* > 0.05.

A number of potential Slam homologs identified in this study were found in bacterial species that colonize the upper respiratory tract of mammals. Human respiratory tract bacteria that contain Slam sequences include *N. meningitidis, M. catarrhalis* and the HACEK (*Haemophilus, Aggregatibacter, Cardiobacterium, Eikenella, Kingella*) group of bacteria (Nørskov-Lauritsen, 2014). Slam sequences were also identified in bacteria that colonize the upper respiratory tract of cattle (*Moraxella bovis, Mannheimia haemolytica*, and *Histophilus somni*) and pigs (*Actinobacillus pleuropneumoniae*). Many of these species have been reported to contain transferrin-binding surface lipoproteins that are homologs of TbpB in *N. meningitidis* (Gray-Owen and Schyvers, 1996). Not surprisingly, two TbpB homologs in *M. catarrhalis* (Yu and Schryvers, 1993) and *H. influenzae* (Gray-Owen et al., 1995) were found to be adjacent to putative Slam genes (Figure 2A). Both *M. catarrhalis* and *H. influenzae* are human pathogens and their TbpBs have been previously shown to bind to human transferrin. The presence of TbpB genes adjacent to a Slam gene in their genome strongly suggests that these bacteria use a Slam-dependent translocation system to deliver TbpBs to the bacterial cell surface.

To confirm that these TbpBs depend on their adjacent Slam for translocation to the cell surface, we introduced the gene encoding TbpB in *M. catarrhalis* in laboratory strains of *E. coli* with and without its neighboring Slam. To monitor the expression of *M. catarrhalis* TbpB, we introduced a FLAG-tag at the C-terminus of TbpB. We labeled the cells with biotinylated human transferrin or α-Flag antibodies (Figure 2B). Flow cytometry was used to quantify the amount of *M. catarrhalis* TbpB on the surface of the cell. An increase in *M. catarrhalis* TbpB was observed only in the presence of its neighboring Slam and was quantified using mean fluorescence intensity (MFI) (Figure 2C). Western blots with α-Flag and by α-His antibodies were used to test the expression of *M. catarrhalis* TbpB & Slam respectively (Figure 2C, lower panel). *M. catarrhalis* TbpB was robustly displayed on the surface of *E. coli* in the presence of Slam confirming that this TbpB homolog also uses a Slam-dependent system to reach the cell surface.

To verify that the FLAG-tag did not affect the Slam-dependent display of *M. catarrhalis* TbpB, we also tested the surface display of *M. catarrhalis* TbpB without the FLAG-tag in laboratory strains of *E. coli*. We labeled the cells using biotinylated human transferrin to test the functional display of the TbpB. Flow cytometry was used to quantify the amount of TbpB located on the surface of the cell. Similar to the results obtained for the FLAG-tagged construct, an increase in signal was obtained only in the presence of the neighboring Slam gene suggesting that the FLAG-tag does not affect translocation (Figure 2D). Additionally, we also tested the surface display of *H. influenzae* TbpB with or without its neighboring Slam using biotinylated human transferrin. In this case, a signal increase was obtained on the surface of *E. coli* in the presence of Slam, however the signal was weaker compared to *M. catarrhalis* TbpB. Westerns blot analysis using α-His antibody suggested that *M. catarrhalis* Slam (Figure 2D, lower panel) is expressed much more strongly in comparison to *H. influenzae* Slam (Figure 2E, lower panel), which may contribute to the lower signal obtained for *H. influenzae* TbpB.

### Predicted SLP genes are found adjacent to slam genes in a number of gram-negative bacteria

**Figure 3:**
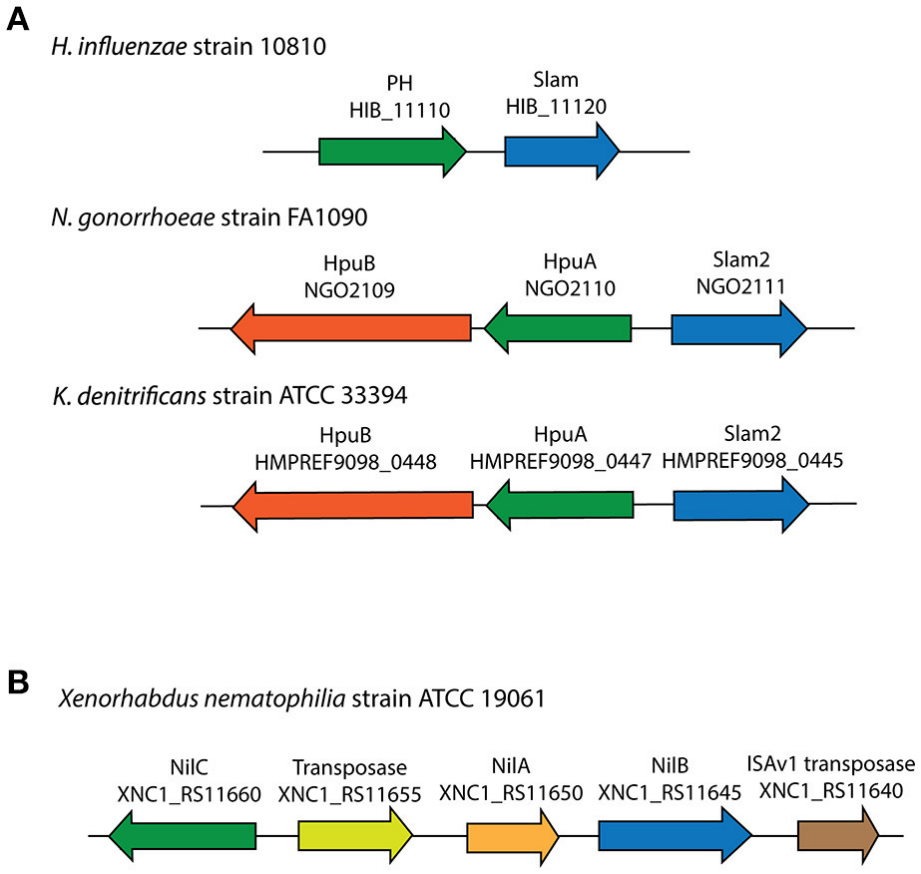
**Slam related gene clusters identified in this study with known lipoproteins**. **(A)** Three SLP-containing Slam gene clusters were found in *Neisseria gonorrhoeae, Kingella denitrificans* and *Haemophilus influenzae*. **(B)** Lipoprotein-containing Slam gene cluster in *Xenorhabdus nematophilia*.

For 353 of the 832 Slam related genes identified, a list of neighboring genes was analyzed using InterProScan (Jones et al., 2014) to identify Slam related gene clusters that also contain lipoprotein-encoding genes. From our analysis, 185 of the 353 (~52% of the clusters examined) Slam related genes contained lipoprotein-encoding genes in their gene clusters. This list of putative lipoproteins contained three experimentally confirmed SLPs (Figure 3A). These include two HpuA homologs in *Neisseria gonorrhoeae* and *Kingella denitrificans* that were functionally and structurally characterized by Wong et al. (2015). We also identified a putative human factor H binding protein from *H. influenzae*, referred to as protein H (PH) (Fleury et al., 2014) that has sequence homology to the Slam-dependent SLPs in *N. meningitidis*.

The 185 predicted lipoproteins were found in 129 different species distributed throughout *Proteobacteria*. One such lipoprotein was identified in the nematode pathogen *Xenorhabdus nematophila* that has not been previously shown to contain SLPs (Figure 3B). This lipoprotein, named NilC has been previously shown to be lipidated *in vivo*, present in the outer membrane and is important for host colonization (Cowles and Goodrich-Blair, 2004). The gene encoding NilB, a putative Slam homolog, is present next to the gene encoding NilC, and is shown to be required for host colonization (Bhasin et al., 2012). Our analysis suggests that NilC is a surface lipoprotein (SLP) that is dependent on the Slam homolog, NilB, for surface display.

Studying other genes in the Slam related gene clusters, we uncovered that 120 of the 185 clusters also possessed genes that encode proteins annotated as TonB-dependent receptors (TonBDR). This is not surprising as in *Neisseria* sp., three Slam-dependent SLPs (TbpB, LbpB, and HpuA) work in conjunction with a TonBDR to acquire iron (Hooda et al., 2017). The presence of TonBDRs gene in proximity to these putative SLPs supports their potential roles in nutrient acquisition. Furthermore, a small subset of Slam gene clusters (nine) contained multiple lipoprotein-encoding genes suggesting that they may be responsible for the display of multiple target SLPs.

### A putative SLP gene in *Pasteurella multocida* is displayed on the surface of *E. coli* in a slam-dependent manner

**Figure 4:**
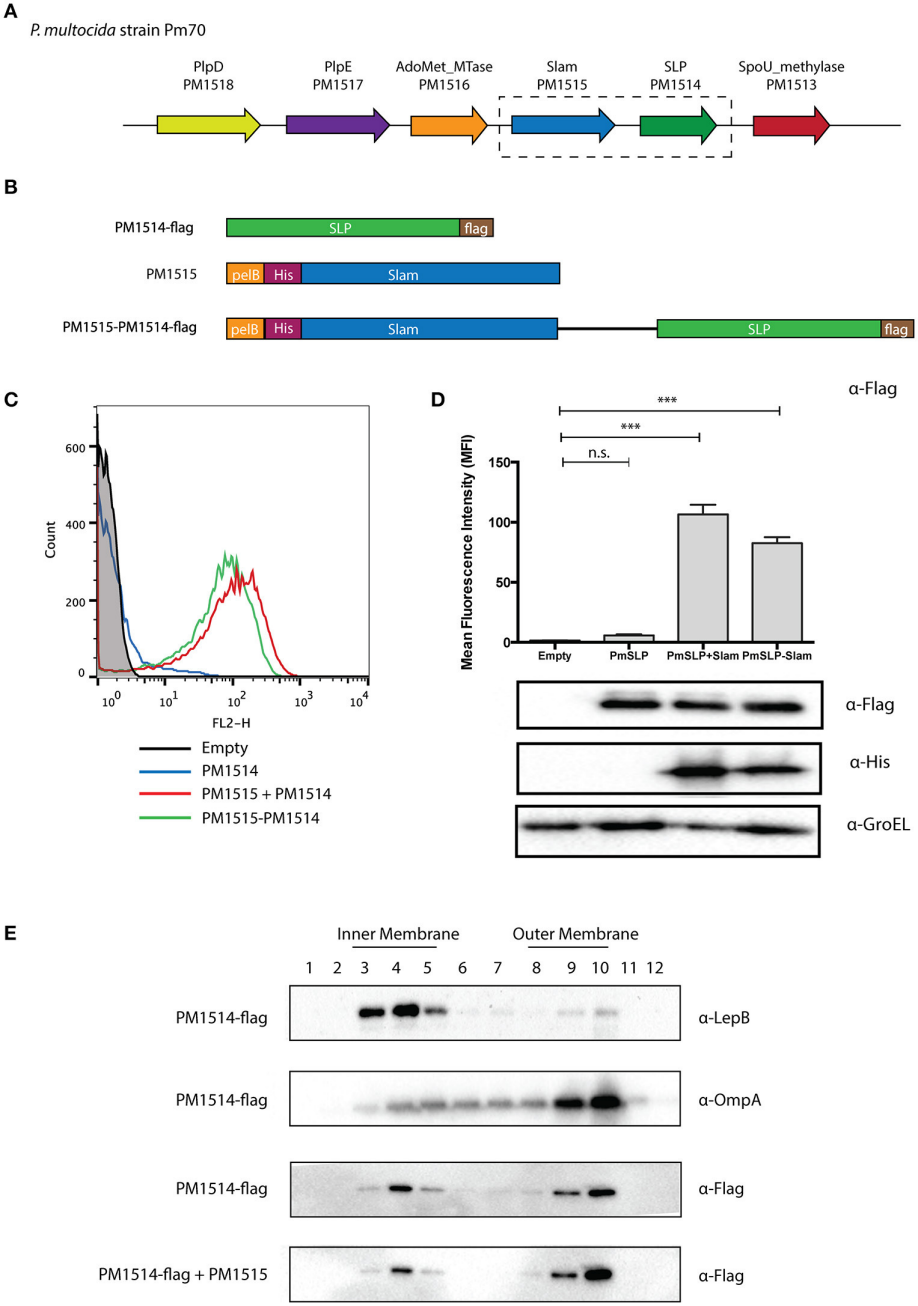
**Identification of a putative Slam-dependent surface lipoprotein in *Pasteurella multocida*. (A)** Slam gene cluster in *P. multocida* strain Pm70. PM1515 (shown in blue) was identified as a putative Slam homolog in our bioinformatics search. PM1514 (shown in green) was annotated as a hypothetical protein with a predicted signal peptidase II cleavage site located within a putative lipobox motif. **(B)** *P. multocida* constructs utilized in an *E. coli* reconstituted translocation assay. To investigate if PM1514 is a Slam-dependent SLP, we cloned the following: PM1514 with a C-terminal FLAG-tag (PM1514-flag), PM1515 with an N-terminal His-tag and pelB signal sequence, and PM1515-PM1514-flag construct with both PM1515 and PM1514 region. **(C)** Flow cytometry profiles of *P. multocida* constructs. The 3 constructs were expressed in *E. coli* C43 (DE3) cells and labeled with α-Flag antibody and a secondary antibody directed against mouse IgG and linked to R-PE. Flow cytometry profiles of empty plasmid (black), PM1514-flag (blue), PM1515+PM1514-flag (red) and PM1515-PM1514-flag (green) are shown. **(D)** Mean fluorescence intensity plots for *P. multocida* constructs. Surface PM1514 was quantified using mean fluorescence intensity (MFI). Statistical significance was determined using one-way ANOVA. ^***^ *p* ≤ 0.001, n.s. *p* > 0.05. **(E)** Localization of *P. multocida* SLP using sucrose density ultracentrifugation. To test the localization of *P. multocida* SLP in *E. coli*, cells expressing PM1514-flag with or without PM1515 were harvested. Cell membranes were then isolated and layered on a sucrose gradient to separate the inner and outer membrane. Westerns blots were performed on different fractions with α-Flag, α-LepB, and α-OmpA antibodies to detect PmSLP, LepB (inner membrane control) and OmpA (outer membrane control) respectively.

To further confirm the hypothesis that we have identified a large family of Slam-dependent SLPs, we sought to characterize the surface display of some of the remaining lipoproteins that were identified in this study for which no other functional data could be found. One such predicted lipoprotein was found in *P. multocida*, a zoonotic pathogen that resides in the normal respiratory microbiota of mammals. We identified the gene of the putative Slam (PM1515) adjacent to the predicted lipoprotein (PM1514) in all the sequenced strains of *P. multocida* (Figure 4A). The Slam displayed 32% identity to *N. meningitidis* Slam1 while the putative SLP showed no sequence similarity to any of the known Slam-dependent neisserial SLPs. Interestingly, the Slam gene cluster also included two other SLPs, PM1517 (PlpD), and PM1518 (PlpE) that have been investigated as potential vaccine antigens against *Pasteurella* infections (Nardini et al., 1998; Wu et al., 2007).

To test if this *pm1514* gene encoded a Slam-dependent SLP, we cloned the predicted Slam and lipoprotein genes into *E. coli* expression vectors (Figure 4B). We transformed *E. coli* cells with FLAG-tagged *P. multocida* lipoprotein and Slam, and used the FLAG-epitope to detect the SLP on the surface. As predicted, we saw an increase in FLAG signal upon expression of Slam as seen in flow cytometry profiles (Figure 4C) and quantified in mean fluorescence intensity (MFI) plots (Figure 4D). Furthermore, to confirm the translocation pathway used by the *P. multocida* SLP, we examined its outer membrane localization in the presence or absence of Slam by sucrose density ultracentrifugation (Figure 4E). Collectively, these findings suggest that PM1514 is a putative Slam-dependent SLP and that many other Slam-dependent SLPs on our list (Supplementary Data 2) likely use a similar translocation pathway as *N. meningitidis* TbpB to reach the surface (Hooda et al., 2016).

### Comparison of putative SLP proteins revealed a conserved structural domain

**Figure 5:**
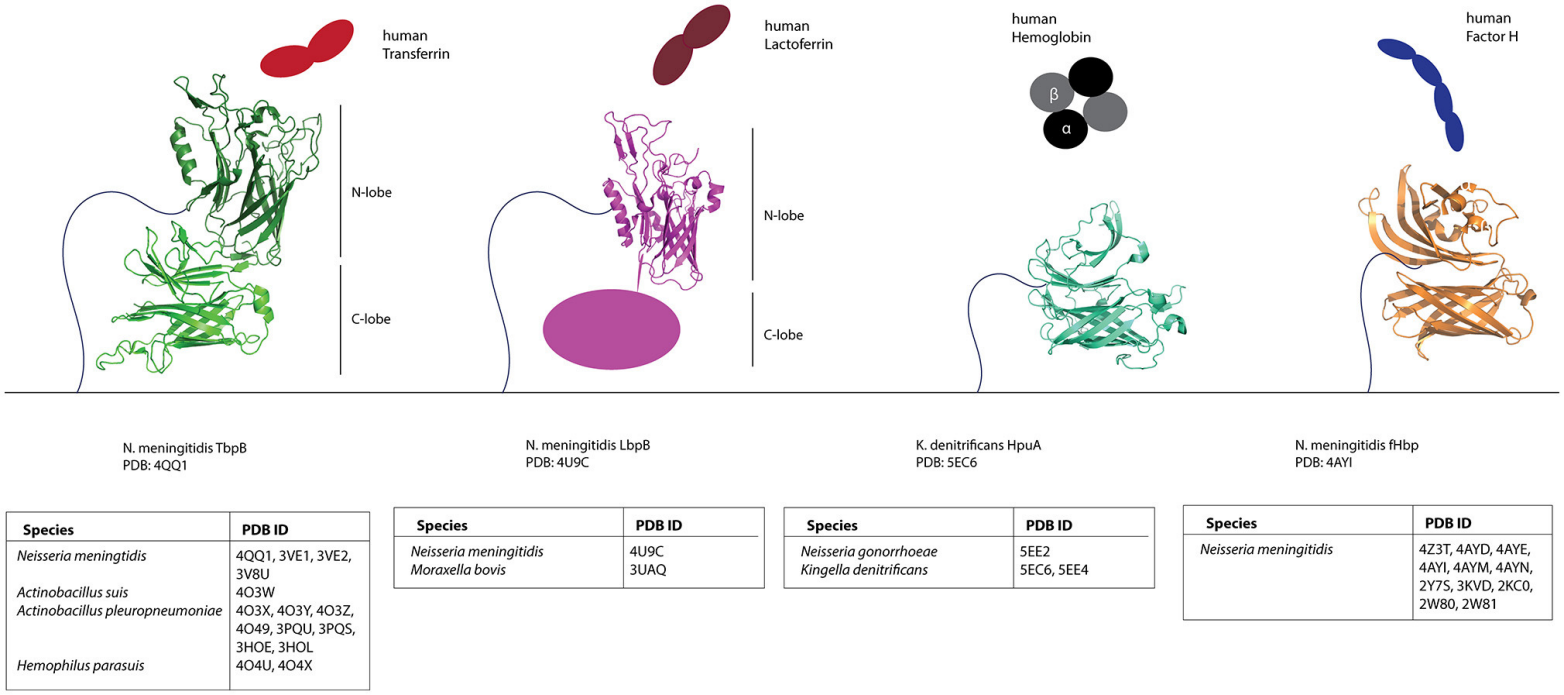
**Structures of known Slam-dependent SLPs**. Structures of TbpB (green), LbpB N-lobe (magenta) HpuA (cyan) and fHbp (orange) are shown. The binding partner for each of the SLPs [human transferrin (red), lactoferrin (brown), hemoglobin (black), and Factor H (blue)] is also shown. Table below lists the accession numbers for all Slam-dependent SLP structures deposited to the Protein Data Bank (PDB). Primary references for TbpB (Moraes et al., 2009; Calmettes et al., 2011, 2012; Noinaj et al., 2012; Adamiak et al., 2015; Frandoloso et al., 2015), LbpB N-lobe (Arutyunova et al., 2012; Brooks et al., 2014), HpuA (Wong et al., 2015), and fHbp (Cantini et al., 2009; Schneider et al., 2009; Cendron et al., 2011; Scarselli et al., 2011; Johnson et al., 2012; Konar et al., 2015) structures are also included.

With this dataset of putative Slam-dependent SLPs, we were interested in identifying structural features that are shared by this family of proteins. Structures for four neisserial Slam-dependent SLPs have been solved by X-ray crystallography and NMR (Figure 5). While these proteins share no sequence similarity, they do share a protein domain composed of a flexible handle domain followed by an eight-stranded barrel domain. *N. meningitidis* TbpB and LbpB contain two lobes of the conserved domain, while fHbp and HpuA contain only one lobe.

To gain insight into the structure of Slam-dependent SLPs, we compared the predicted domains (InterProScan) on our list of putative Slam-dependent SLPs. We found that 58 contain either a lipoprotein GNA1870-related (InterPro signature: IPR014902) or a solute-binding protein, TBP-like (InterPro signature: IPR001677) domain. These domains are found on fHbp and TbpB respectively, both of which are Slam-dependent SLPs, and contain an eight-stranded beta barrel at their C-terminus. Further, 127 were predicted to encode a PagP-beta barrel (InterPro signature: IPR011250). PagP is an outer membrane enzyme that forms an eight-stranded barrel and is involved in catalyzing palmitate transfer from a phospholipid to a glucosamine unit of lipid A (Cuesta-Seijo et al., 2010). While PagP is an integral outer membrane protein, several soluble proteins are also predicted to contain an eight-stranded PagP-beta barrel. Most of the predicted Slam-adjacent lipoproteins also had a variable N-terminal region preceding the eight-stranded barrel that could form a handle-like domain seen in the representative three-dimensional structures of Slam-dependent SLPs.

## Discussion

With the increase in the number of bacterial genomic sequences, it has become evident that surface lipoproteins (or SLPs) are widespread in Gram-negative bacteria. One family of SLPs is characterized by a common structural architecture composed of an eight-stranded beta-barrel domain and a beta rich handle domain (TbpB, LbpB, HpuA, and fHbp). These proteins require a member of a unique family of proteins to traverse the outer membrane named Slam (Hooda et al., 2016). While we still do not know the exact role played by Slams, these proteins are specific for TbpB-like SLPs and may represent a novel class of outer membrane translocon or chaperone dedicated to the transport of SLPs from the inner leaflet of the outer membrane to the surface.

In this study, we performed a bioinformatic analysis of putative Slam homologs and identified a number of Slam-dependent SLPs in many different species of *Proteobacteria*. To our knowledge, this is the first systematic study to look at the distribution of SLPs in different Gram-negative bacteria. Previous attempts to look at TbpB-like lipoproteins have been stymied by the degree of variation that is found in these proteins. Since Slam sequences are more conserved, we were able to identify a large number of SLP homologs owing to the genetic linkage between Slams and TbpB-like SLPs. We have extended the number of bacteria that are now predicted to possess SLPs and also provide a framework to systematically search for these proteins. Based on this work, Slam-dependent SLPs represent the largest sub-family of SLPs reported thus far. However, our approach also has some limitations. To date, we have only identified genes for putative SLPs located in the immediate vicinity of genes for Slams. Using this method, we would have overlooked many known Slam-dependent SLPs in pathogenic *Neisseria* sp., which are transported in a Slam1-dependent manner but are encoded by genes not located in the vicinity of the gene for Slam1 (e.g., NmTbpB and NmLbpB). Therefore, our list of Slam-dependent SLPs is certainly incomplete; the bacterial species containing Slam homologs but no Slam-adjacent lipoproteins may also contain SLPs elsewhere in the genome.

Our analysis allowed the identification of a previously uncharacterized protein PM1514 in *Pasteurella multocida* as a putative SLP. Using localization assays we confirmed PM1514 is present in the outer membrane but is only detected on the surface of *E. coli* when co-expressed with the putative Slam PM1515. Taken together, these findings suggest that PM1515 is a Slam homolog and PM1514 is a Slam-dependent SLP. Additional work is required to further investigate this putative SLP, including a ^3^H-palmitoyl labeling assay to confirm the lipidation of PM1514 in both *E. coli* and *P. multocida*. Flow cytometry and proteinase K shaving assay should be performed in *P. multocida* to test the surface display of PM1514 in its endogenous host. PM1514 is found in most sequenced strains of *P. multocida*, indicating that it may play an important role in the survival of *P. multocida* in its host organisms.

In our present search, we identified that Slam related sequences are found in *Proteobacteria* and not found in *Bacteroides, Borrelia*, and *Campylobacter*. Interestingly, we investigated the gene neighborhoods of four other known SLPs namely, JlpA in *Campylobacter* (Jin et al., 2001), HmuY (Wójtowicz et al., 2009) and SusD (Shipman et al., 2000) in *Bacteroides* and OspA/C in *Borrelia* (Schulze et al., 2010). Upon inspection none of these SLPs showed genetic linkage with any known/predicted outer membrane protein (data not shown) nor were any Slam related sequences found in *Bacteroides* or *Borrelia*. Coupled with the mechanistic studies completed on the SLP translocation pathways for *Bacteroides* (Lauber et al., 2016) and *Borrelia* (Chen and Zückert, 2011), it is likely that other translocation systems are used by *Bacteroides* and *Borrelia* to facilitate translocation of these SLPs to the cell surface.

Using the list of putative Slam-dependent SLPs, we have found that all these proteins contain a predicted eight-stranded barrel domain. All Slam-dependent SLPs contain either one or two copies of this barrel domain. Hence, we predict that the barrel domain may contain the translocation motif that is recognized by Slam. However, further experiments are required to tease apart the translocation motif present on these SLPs. Toward this end, the dataset generated by this study will be a valuable tool to identify secretion motif that is common amongst Slam-dependent SLPs.

The ability of Slam proteins to robustly potentiate the display of TbpB-like SLPs from a diverse set of bacteria in *E. coli* provides further evidence of their direct involvement in translocation. While more work is required to understand the mechanism of Slam function, the work done so far shows the efficacy of using Slam as a system to deliver proteins to the surface of Gram-negative bacteria and has potential applications in development of bacterial surface display technology (Ståhl and Uhlén, 1997). Taken together with our previous study (Hooda et al., 2016), our work suggests that TbpB-like SLPs and Slams form a “plug-and-play” cassette, reminiscent of the two-partner secretion systems (TPSS) or Type Vb secretion system (Jacob-Dubuisson et al., 2013). To date, we believe that Slams are specific for the delivery of lipidated proteins to the surface of Gram-negative bacteria. However, it will be interesting to see if they represent a more generalized secretion system. Finally, this study furthers the argument that SLPs are an important and yet under-appreciated family of proteins and their investigation may lead to identification of novel mechanisms utilized by many different bacteria to interact with their environmental surroundings and/or their hosts.

## Author contributions

YH and TM designed the experiments and wrote the manuscript. YH did the bioinformatics analysis. YH and CL. performed the experiments. YH, CL, and TM. analyzed the data.

### Conflict of interest statement

The authors declare that the research was conducted in the absence of any commercial or financial relationships that could be construed as a potential conflict of interest. However, a patent titled “Slam polynucleotides and polypetides and uses thereof” CA2017050160 has been filed. The reviewer FJD and handling Editor declared their shared affiliation, and the handling Editor states that the process nevertheless met the standards of a fair and objective review.
