# Supplementary material for: Identification of a Large Family of Slam-Dependent Surface Lipoproteins in Gram-Negative Bacteria
Source: Front Cell Infect Microbiol. 2017 May 31;7:207. doi: 10.3389/fcimb.2017.00207 (PMC5449769; doi:10.3389/fcimb.2017.00207)
Supplement: Supplementary file 1 [file DataSheet1.DOCX]

Supplementary Material

**Identification of a Large Family of Slam-dependent Surface Lipoproteins in Gram-negative Bacteria**

Yogesh Hooda, Christine Chieh-Lin Lai, Trevor F. Moraes^*^

*** Correspondence:** Trevor F. Moraes: trevor.moraes@utoronto.ca

# Supplementary Data

**Supplementary data 1: List of Slam homologs identified in this study.** Bacterial species with Slam homologs are listed with the number of Slam homlogs in each species. Slams selected for further investigation are highlighted in grey.

**Supplementary data 2: Gene neighborhood analysis for 353 Slam homologs.** Neighboring genes for select Slams were manually curated and domain signatures were retrieved. IntroProScan and Pfam domain databases were used to identify proteins with a lipobox motif. Based on the analysis, the Slam gene clusters were divided into: Clusters with no lipoprotein gene (0), Clusters with a lipoprotein gene (1), Clusters with a lipoprotein and a TonBDR gene (2) and Clusters with insufficient sequence information (99). In total, 185 Slam gene clusters were found to contain putative lipoproteins.

# Supplementary Figures and Table

# Supplementary Table 1: List of reagents used in this study

|  | **Description** | **Reference** |
| --- | --- | --- |
| **Strains:** |  |  |
| *Escherichia coli MM294* | *F-, glnX44(AS), LAM-, rfbC1, endA1, spoT1, thiE1, hsdR17, creC510* | Yale CGSC #6315 |
| *Escherichia coli C43* | *F– ompT gal dcm hsdSB(rB- mB-)(DE3)* | C43 reference |
|  |  |  |
| **Genomes:** |  |  |
| *Moraxella catarrhalis O35E* | Strain obtained from Dr. Anthony Schryvers | (Mathers et al., 1997) |
| *Haemophilus influenzae 86-028NP* | Strain obtained from Dr. Scott Gray-Owen | (Harrison et al., 2005) |
| *Pasteurella multocida h48* | Strain obtained from Dr. Anthony Schryvers | (Ogunnariwo and Schryvers, 2001) |
|  |  |  |
| **Plasmids:** |  |  |
| pET26b | *E.coli* expression vector | Addgene, 69862-3 |
| pET26 McSlam | pET26 containing pelB signal peptide, 7xHis and mature Mc Slam | This study |
| pET26 HiSlam | pET26 containing pelB signal peptide, 7xHis and mature Hi Slam | This study |
| pET26 PmSlam | pET26 containing pelB signal peptide, 7xHis and mature Pm Slam | This study |
| pET52b | *E.coli* expression vector | Novagen, 71554-3 |
| pET52b TbpB | pET52b containing Nm TbpB with endogenous signal sequence | This study |
| pET52b HpuA | pET52b containing Nm HpuA with endogenous TbpB signal sequence | This study |
| pET52b McTbpB | pET52b containing Mc TbpB with endogenous signal sequence | This study |
| pET52b McTbpB-flag | pET52b McTbpB with a C-terminal flag tag | This study |
| pET52b HiTbpB | pET52b containing Hi TbpB with endogenous signal sequence | This study |
| pET52b PmSLP-flag | pET52b containing Pm SLP with endogenous signal sequence | This study |
| pET53b PmSLP-flag-Slam | pET52b containing PM1514with endogenous signal peptide and PM1515 | This study |
|  |  |  |
| **Antibodies:** |  |  |
| α-LepB | Rabbit polyclonal antibodies were obtained from Dr. Jan Willem de Gier | (de Gier et al., 1998) |
| α -OmpA | Rabbit polyclonal antibodies were obtained from Dr. Jan Willem de Gier | (Schlegel et al., 2013) |
| α-GroEL | Rabbit polyclonal antibodies were obtained from Dr. Walid Houry | (Houry et al., 1999) |
| α-Flag | Mouse anti-Flag monoclonal antibody | Sigma, F3040-5MG |
| α-His | Mouse anti-6x His monoclonal antibody | Pierce, MA121315 |
| Bio-hTf | Biotinylated human transferrin | Sigma, T3915-5MG |
| PE-Streptavidin | R-Phycoerythrin-Streptavidin | Cederlane, 016-110-084 |
| PE-mouse IgG | Immunopure PE polyclonal goat anti-mouse IgG | Pierce, PI31861 |
| HRP-rabbit IgG | Goat anti-rabbit IgG antibody conjugated to horseradish peroxidase (HRP) | Cell Signalling, #7074S |
| HRP-mouse IgG | Goat anti-mouse IgG antibody conjugated to horseradish peroxidase (HRP) | Pierce, PI31430 |

**References:**

de Gier, J. W., Scotti, P. A., Sääf, A., Valent, Q. A., Kuhn, A., Luirink, J., et al. (1998). Differential use of the signal recognition particle translocase targeting pathway for inner membrane protein assembly in Escherichia coli. *Proc. Natl. Acad. Sci. U. S. A.* 95, 14646–14651.

Harrison, A., Dyer, D. W., Gillaspy, A., Ray, W. C., Mungur, R., Carson, M. B., et al. (2005). Genomic sequence of an otitis media isolate of nontypeable Haemophilus influenzae: comparative study with H. influenzae serotype d, strain KW20. *J. Bacteriol.* 187, 4627–4636. doi:10.1128/JB.187.13.4627-4636.2005.

Houry, W. A., Frishman, D., Eckerskorn, C., Lottspeich, F., and Hartl, F. U. (1999). Identification of in vivo substrates of the chaperonin GroEL. *Nature* 402, 147–154. doi:10.1038/45977.

Mathers, K. E., Goldblatt, D., Aebi, C., Yu, R., Schryvers, A. B., and Hansen, E. J. (1997). Characterisation of an outer membrane protein of Moraxella catarrhalis. *FEMS Immunol. Med. Microbiol.* 19, 231–236. doi:10.1111/j.1574-695X.1997.tb01092.x.

Ogunnariwo, J. A., and Schryvers, A. B. (2001). Characterization of a Novel Transferrin Receptor in Bovine Strains of Pasteurella multocida. *J. Bacteriol.* 183, 890–896. doi:10.1128/JB.183.3.890-896.2001.

Schlegel, S., Rujas, E., Ytterberg, A. J., Zubarev, R. A., Luirink, J., and de Gier, J.-W. (2013). Optimizing heterologous protein production in the periplasm of E. coli by regulating gene expression levels. *Microb. Cell Factories* 12, 24. doi:10.1186/1475-2859-12-24.
